# Protozoan Intestinal Parasitic Infection in Patients with Hematological Malignancies

**DOI:** 10.3390/jcm11102847

**Published:** 2022-05-18

**Authors:** Aleksandra Łanocha, Natalia Łanocha-Arendarczyk, Dominika Wilczyńska, Barbara Zdziarska, Danuta Kosik-Bogacka

**Affiliations:** 1 Department of Hematology with Bone Marrow Transplantation Unit, Pomeranian Medical University in Szczecin, 71-242 Szczecin, Poland; aleksandra.lanocha@pum.edu.pl (A.Ł.); zdzb@pum.edu.pl (B.Z.); 2 Department of Biology and Medical Parasitology, Pomeranian Medical University in Szczecin, 70-111 Szczecin, Poland; dwilczynska23@gmail.com; 3 Independent Laboratory of Pharmaceutical Botany, Pomeranian Medical University in Szczecin, 70-111 Szczecin, Poland; danuta.kosik.bogacka@pum.edu.pl

**Keywords:** *Blastocystis* spp., *Cryptosporidium* spp., *Entamoeba coli*, *Giardia intestinalis*, hematological malignancies

## Abstract

The aim of this study was to evaluate the frequency of gastrointestinal protozoan infection in patients with hematological malignancies (HMs) undergoing intensive hemato-oncological treatment and to determine the influence of certain biological factors on the incidence of intestinal parasite infection. Stool samples were collected from hematological malignancy patients (*n* = 50) hospitalized at the Department of Hematology and Transplantology of the Pomeranian Medical University in Szczecin. The control group consisted of 50 healthy participants. We used a direct smear examination and a commercial immunoenzymatic test. Intestinal protozoans were detected in 16% of patients with hematological malignancies and in 6% of individuals in the control group. In stool samples from patients with HM, cysts of *Giardia intestinalis* (2%), oocysts of *Cryptosporidium* spp. (10%), vacuolar forms of potentially pathogenic *Blastocystis* spp. (2%), and cysts of nonpathogenic *Entamoeba coli* (2%) were found. *Cryptosporidium* spp. and *Giardia intestinalis* coproantigens were detected in 5 (10%) and 1 (2%) patients with HM, respectively. In three participants from the control group, vacuolar forms of *Blastocystis* spp. were found. In the patients with HM, a significantly higher prevalence of intestinal parasite infection was found in individuals working in the garden without protective gloves and those in contact with animals. In patients with hematological malignancies, intestinal parasites should be excluded, even during intensive chemotherapy treatment.

## 1. Introduction

Hematological malignancies (HMs) are a heterogeneous group of diseases of diverse incidence, prognosis and etiology, characterized by an uncontrolled malignant proliferation of hematopoietic cells. According to recent data, HM are estimated to comprise approximately 6.5% of all cancers worldwide in 2012 [1]. The World Health Organization predicts that the number of HM cases will increase by approximately 48% in less developed countries in 2030 compared to 2012 [1].

Hematological malignancies and hemato-oncological treatments, such as chemotherapy, immunotherapy, biological treatment and hematopoietic cell transplantation, are the causes of secondary immunodeficiencies [2]. They can lead to decreases in humoral immunity, cellular immunity, complement abnormalities and neutropenia and impairment of nonspecific immunity, which significantly increase the risk of infection [3,4].

An increase in the risk of certain parasitic infections has also been found with a reduction in CD4 lymphocyte count in the peripheral blood of the host [5,6]. In addition, parasitosis in immunocompromised patients can lead to opportunistic diseases with severe symptoms [5,6,7]. Opportunistic infections may be associated with reinvasion, polyinfection, predisposition to autoinfection and a high risk of the parasite acquiring highly pathogenic characteristics [8]. The most common parasites in such patients are *Giardia intestinalis* and intestinal coccidia (i.e., *Cryptosporidium* spp., *Isospora belli* and *Cyclospora cayetanensis*). In recent years, *Blastocystis* spp. and *Microsporidia* spp. have been reported as emerging pathogens in immunocompromised hosts [9]. *Strongyloides stercoralis* is frequently found in patients with human T-cell lymphotropic virus type 1 infection, a virus associated with adult T-cell leukemia/lymphoma [10].

In our previous research, we noted respiratory failure associated with ascariasis in patient with AML (*acute myeloid leukemia)* [7]. Some authors observed a higher prevalence of *Blastocystis* spp. and *Giardia intestinalis* infection in patients with acute lymphoblastic leukemia (ALL) [11].

Most studies on the prevalence of intestinal parasite in patients with HM were conducted in tropical countries, with a prevalence rate from 12% in patients receiving chemotherapy for HM in Turkey to 69.5% in pediatric patients with hematologic neoplasms in Mexico [12,13,14,15,16,17].

The aim of this study was to evaluate the frequency of gastrointestinal protozoan infection during intensive hemato-oncological treatment of hematological malignancies. Moreover, in order to determine the influence of biological factors on the prevalence of parasites, our study included factors such as age, gender, hematopoietic cell transplantation as well as the presence of intestinal symptoms, and risk factors, such as the consumption of unwashed fruits and vegetables, contact with animals and soil and previous travel to tropical countries. We also analyzed the association between the prevalence of intestinal parasites and the type of hematopoietic malignancy.

## 2. Materials and Methods

The study was approved by the Bioethics Committee of the Pomeranian Medical University (PUM) in Szczecin (KB-0012/54/17). It conformed to the principles outlined in the Declaration of Helsinki as revised in 2008.

### 2.1. Characteristics of the Groups

Stool samples were collected from hematological malignancy patients (*n* = 50) hospitalized in 2017–2018 at the Department of Hematology and Transplantology PUM. The HM group consisted of 17 women (34%) and 33 men (66%). The mean age of these patients was 59.4 ± 14.7 years (61.8 ± 11.9 years for women and 58.1 ± 16.0 years for men). The HM group consisted of patients diagnosed with malignancies such as acute myeloblastic leukemia (AML; 30%), diffuse large B-cell lymphoma (DLBCL; 22%), plasma cell myeloma (PCM; 8%), acute lymphoblastic leukemia (ALL; 6%), myelodysplastic syndrome (MS; 6%), Burkitt’s lymphoma (BL; 4%) and myelodysplastic/myeloproliferative neoplasm (MPN; 4%). Patients in the HM group received intensive hemato-oncological treatment, and 43 (86%) patients additionally received steroid therapy. Subjects who had taken antidiarrhea compounds, barium, bismuth or mineral oil (≤3 weeks) were excluded. The control group (CG) consisted of 50 healthy subjects (28 women—56%; 22 men—44%). The mean age of the CG patients was 50.94 (51.4 ± 22 years for women; 49.4 ± 21 years for men).

The individuals from the HM and CG groups were divided into four age groups: ages 20–40 (A1; *n* = 6 and *n* = 21); 41–60 (A2; *n* = 14 and *n* = 10); 61–80 (A3; *n* = 28 and *n* = 17); >80 (A4; *n* = 2 and *n* = 2). Immunocompetent individuals had no gastrointestinal symptoms and went to the Laboratory of the Department of Biology and Medical Parasitology PUM (LDBMP) for a standard stool parasitological examination. Exclusion criteria for the CG included the presence of chronic and neoplastic diseases, chemotherapy, and the use of immunosuppressive drugs.

### 2.2. Collection of Stool for Testing

Before stool collection, patients had to give their consent for the study and were informed about the rules of stool collection and transportation. Stool from all of the patients were collected in a special disposable container, and in cases where the stool was collected from a hemato-oncological patient, the procedure was conducted under the control of medical staff. Patients completed a questionnaire about their eating habits, hygiene habits, contact with animals and travel history. Additionally, HM patients completed a questionnaire about their disease, the use of immunosuppressive drugs, chemotherapy, transplantation history and coexisting chronic diseases.

### 2.3. Parasitological Stool Examination

Stool samples were examined immediately upon arrival at the parasitology laboratory. Macroscopic examination determined stool consistency (i.e., watery, loose or firm) and, additionally, assessed the presence of mucus, blood, adult worms and proglottids. A direct preparation in normal saline (0.9%), and Lugol’s iodine solution (diluted in 1:5 distilled water) was made from each test sample (~2 g stool). Moreover, stool samples were stained by modified Ziehl–Neelsen staining. Slides were examined at 1000× magnification under a light microscope to investigate the oocyst of *Cryptosporidium* spp.

Developmental forms of intestinal parasites, including cysts and trophozoites of protozoa, eggs, larvae and mature forms of worms, were sought in the feces.

Other structures indicative of pathology were also sought in the feces including macrophages, red blood cells, polymorphonuclear leukocytes (PMNs), Charcot–Leyden crystals, starch grains, meat fibers and fat globules.

Stool samples were also analyzed to determine the presence or absence of *Cryptosporidium* spp. coproantigen using a commercial immunoenzymatic test (ProSpecT *Cryptosporidium* Microplate Assay), according to the manufacturer’s instructions. The test uses monoclonal antibodies for the qualitative detection of *Cryptosporidium*-specific antigen (CSA), which reduces the possibility of cross-reactivity. It has a sensitivity of 97% and a specificity of 100%. The intensity of the reaction product was analyzed visually.

Fecal samples were examined to determine the presence or absence of *G. intestinalis* coproantigen using an immunoenzymatic test (Thermo Scientific™ ProSpecT™ *Giardia* Microplate Assay), according to the manufacturer’s instructions. The test uses a monoclonal antibody for the qualitative detection of *Giardia*-specific antigen (GSA 65). It has a sensitivity of 96% and a specificity of 98%. The intensity of the reaction product was analyzed visually.

### 2.4. Statistical Analysis

StatSoft Statistica v11.0 and Microsoft Excel 2007 were used for statistical analysis. The nonparametric Mann–Whitney U test (*p* < 0.05) was used for comparisons between study groups. Spearman’s rank correlation coefficient (r_s_) was calculated, and its significance was determined to evaluate the relationship between the frequency of infection in patients with intestinal parasites and the age and sex of patients, as well as the co-occurrence of clinical symptoms. The environmental exposure of the HM and CG patients was also analyzed.

## 3. Results

The presence of intestinal protozoan was detected in eight (16%) patients with HM and in three (6%) individuals in the control group (Table 1). In patients with HM, the prevalence of intestinal parasites was significantly higher than for participants in the CG (*p* < 0.05). The highest occurrence of protozoan parasites (4%) was noted in patients with PCM and DLBCL.

**Figure 1:**
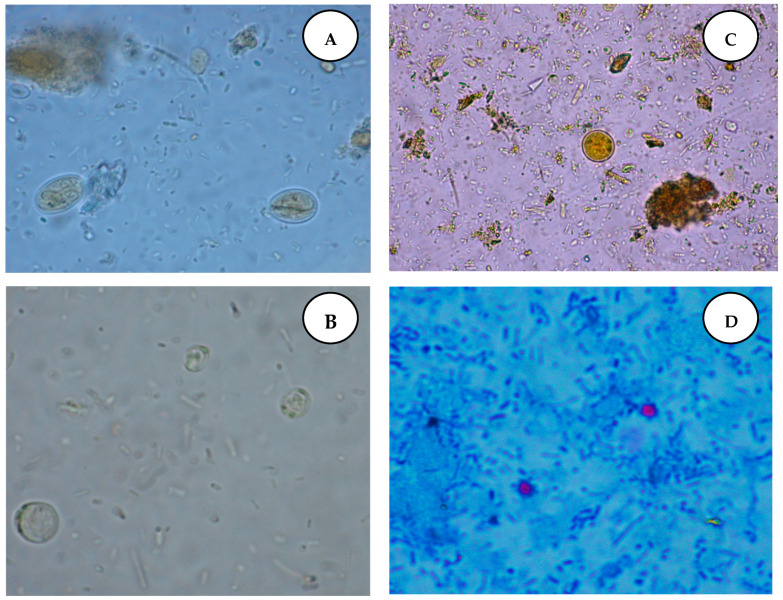
Intestinal parasites identified in stool samples from hematological malignancy patients: (**A**) cyst of *Giardia intestinalis*; (**B**) vacuolar forms of *Blastocystis* spp.; (**C**) cyst of *Entamoeba coli* in a direct smear with Lugol solution, ×1000; (**D**) oval oocysts of *Cryptosporidium* spp. in a direct smear (modified Ziehl–Neelsen staining, ×1000).

In the stool samples of patients with HM, we found cysts of *G. intestinalis* (*n* = 1; 2%), oocysts of *Cryptosporidium* spp. (*n* = 5; 10%), vacuolar forms of potentially pathogenic *Blastocystis* spp. (*n* = 1; 2%) and cysts of nonpathogenic *Entamoeba coli* (*n* = 1; 2%) (Figure 1). *Cryptosporidium* spp. and *G*. *intestinalis* coproantigens were detected in 5 (10%) and 1 (2%) patients with HM, respectively. The three infected patients from the CG had vacuolar forms of *Blastocystis* spp. (<5 parasites per high-power field). The presence of undigested starch grains was noted in four immunocompetent patients (10.3%), and the presence of Charcot–Leyden crystals in the stool was noted in one patient (2.6%). In eight (16%) patients with HM, the parasitological stool contained undigested starch. Patients with the confirmed presence of *Cryptosporidium* spp. coproantigens had leukocytes and blood in their stool, and the stool was watery.

### 3.1. Clinical Cases

*Cryptosporidium* spp. were found in a 66 year old female patient with plasma cell myeloma, who initially presented with a predominant IgG lambda. The patient also had hypertension, diabetes mellitus and ischemic heart disease and was treated with hemodialysis. Laboratory tests revealed normocytic anemia, hypergammaglobulinemia and high creatinine levels. The patient qualified for treatment with Velcade (bortezomib) and dexamethasone. She worked as a farmer with a history of working in the garden without protective gloves and had contact with animals including a dog and cat.

*Cryptosporidium* spp. was also found in a 46 year old patient diagnosed with DLBCL. The patient underwent a rectal swab and stool collection for *Clostridium difficile* infection, and the results were negative. The patient reported a history of gardening without protective gloves and had contact with animals including a dog. *Cryptosporidium* spp. infection was found in a 61 year old patient diagnosed with AML, following a surgery for clear cell renal cell carcinoma, suffering from gallbladder stones and hypertension. The patient was treated with intensive chemotherapy according to the protocol of the Polish Adults Leukemia Group (PALG). He had a history of working in the garden without protective gloves and had contact with animals including a dog and pigeons.

*Cryptosporidium* spp. were also found in a 65 year old IgG-kappa multiple myeloma female patient treated with broadspectrum antibiotic therapy for pneumonia. During hospitalization, the patient remained in serious general condition. The patient had gastrointestinal bleeding and blood was found during the stool examination. Gastroscopy revealed inflammation of the gastric mucosa and polyps. The patient was undergoing chemotherapy and was treated with corticosteroids. She did not have a history of gardening without protective gloves and had no contact with animals.

A 45 year old male patient had an established diagnosis of BL, a history of chemotherapy and radiotherapy for high-grade lymphoma, a left palate and eyeball prosthesis due to the complications from radiotherapy, postoperative treatment for squamous cell carcinoma of the skin in the region of the buttocks and treatment with combined antiretroviral therapy (cART) for HIV. CD4^+^ T-cell count was below 200 cells/μL. The patient had lived in Africa for many years and traveled professionally. The patient had experienced three episodes of malaria. Due to the diagnosis of BL, he qualified for an intense chemotherapy regimen comprising fractionated cyclophosphamide, doxorubicin, vincristine and dexamethasone to which rituximab was added (R-HyperCVAD). CART therapy for the acquired immunodeficiency was conducted under the supervision of the outpatient clinic with good results. The patient was diagnosed with *Cryptosporidium* spp. coproantigen and *Cryptosporidium* oocysts during immunochemotherapy.

*Giardia intestinalis* cysts and *G. intestinalis* antigen were detected in a 61 year old patient with relapsed DLBCL lymphoma treated with German Multicenter Adult ALL Study Group (GMALL) chemotherapy. Oral stomatitis was observed after methotrexate treatment. The patient worked as an automotive refinisher. With a history of diagnosed pinworm, he reported working in the garden without protective gloves and had contact with dogs.

A single *Blastocystis* spp. was observed in the stool of a 61 year old patient with ALL. She was a patient after a hemicolectomy for sigmoid carcinoma with subsequent chemotherapy with a genetic load for colorectal cancer. In addition to the stool parasitological examination, a stool microbiological examination was performed, which showed the presence of gastrointestinal physiological flora with *Escherichia coli* ESBL. The patient was treated with intensive chemotherapy according to the protocol of the PALG (ALL6 PALG >55 years of age) consolidation treatment with methotrexate and dexamethasone. The patient reported a history of gardening without protective gloves and had contact with animals including a cat and a dog.

A 62 year old patient diagnosed with MPN, a welder by occupation, was found to have *Entamoeba coli* cysts. The patient was treated with hydroxycarbamide and corticosteroids. The patient reported a history of working in the garden without protective gloves and had contact with animals including a dog.

Patients with parasites and coproantigens were treated with nitrosoxanide. After 2 weeks, the follow-up stool test showed all samples were negative.

### 3.2. Biological Factors and Parasite Infection

We found no statistically proven relationship between the occurrence of enteric parasites and the gender of the patient. The prevalence of parasites in male (M) and female (F) patients from the HM group were ~4 and 3 times higher (M: 15.2%, 17.7%) than in the CG (M: 5.9%, 4.6%), respectively.

In immunocompetent subjects, the highest prevalence of protozoan parasites was reported in the A1 group and did not exceed 10%. The highest prevalence of intestinal parasite infection in patients undergoing hemato-oncological treatment was found in the age categories A3 and A2 at 22.2% and 13.3%, respectively. The presence of intestinal parasites in the HM group and the CG was not significantly related to age. There was no significant correlation between the consumption of unwashed fruits and/or vegetables, the consumption of unboiled water, raw meats and seafood and the prevalence of intestinal parasites in patients from the HM group or the CG (Table 2).

The comparative analysis of patients with HM showed a significantly higher prevalence of intestinal parasite infections in those who worked in the garden without protective gloves (87.5%) compared to those who protected their hands (12.5%) (U = 12; *p* < 0.05). Moreover, it was shown that hematopoietic cell transplantation in patients with HM had no effect on the presence of intestinal parasites.

In the HM group, we noted a significantly higher prevalence of intestinal parasite infections in patients with animal contact (75%) than in patients without animal contact (25.5%) (U = 10; *p* < 0.05). Infected patients with HM most frequently declared contact with dogs and cats and much less frequently with pigeons, pigs, rabbits and also with birds including hens and/or parrots.

The occurrence of intestinal parasites was not significantly related to travel history. In immunocompromised patients traveling to a different climate zone within the last two years, 16% of patients had travelled outside of Poland to Greece, Israel, Turkey, Malta, and North and East Africa including Egypt, Tunisia and Kenya. Two patients (25%) with a positive travel history had intestinal parasites. A direct stool smear from a 66 year old male, who had travelled to Israel, detected *Blastocystis* spp. In addition, a *Cryptosporidium* spp. coproantigen was found in a 61 year old patient with BL who declared a trip to Kenya two months before the parasitological examination was performed.

There was no statistically significant correlation between the presence of clinical symptoms and parasitic infections between the groups. In patients with HM, the most common symptoms reported were weakness (72%), weight loss (66%) and loss of appetite (56%). Less commonly reported were diarrhea (38%), skin flushing (30%), vomiting (22%), constipation (22%) and difficulty breathing (22%). In patients who were found to have parasites, the co-occurrence of the following symptoms was reported: weight loss and weakness (75%); weight loss and a loss of appetite (62.5%). Less frequently, skin redness (37.5%), diarrhea (25%) and constipation (25%) were reported. No gastrointestinal symptoms were observed in control patients with confirmed *Blastocystis* spp.

## 4. Discussion

The clinical severity and outcome of a parasitic disease often depends on innate and acquired host immunity [18]. Secondary immunodeficiency (SID) has a mixed nature and involves both specific and nonspecific responses. In hematological malignancies, symptoms of immune deficiency increase due to, among other things, the displacement of normal immune cells by tumor cells, the secretion of immunosuppressive factors and immunosuppressive treatment [2]. These host conditions facilitate infection by enteric parasites, among others [5].

In patients with HM, we observed that the prevalence of intestinal parasites was significantly higher than in the immunocompetent group. Enteric parasites (most frequently *Cryptosporidium* spp.) were noted in patients undergoing hemato-oncological treatment for PMC, DLBCL, ALL, AML and BL. Stark et al. [19] stated that non-Hodgkin’s lymphoma (NHL), leukemia and lymphoproliferative disease may be risk factors for cryptosporidiosis. Botero et al. [6] demonstrated that the prevalence of *Cryptosporidium* spp. in patients with AML and chronic lymphocytic leukemia (CLL) did not exceed 4%—moreover, the authors did not find any association between the occurrence of intestinal parasites and the type of immunodeficiency. On the other hand, Bednarska et al. [5] observed a link between micropathogen infections, including *Cryptosporidium* spp., *Giardia* spp., *Blastocystis* spp., microsporidia and immunosuppressed rates. Most parasitic infections were reported in patients with severe second-stage immunodeficiency (6.1%), and in patients with mild or no immunosuppression, it was 0.6% and 2.2%. The prevalence of *Cryptosporidium* spp. in the HM patients in this study was similar to the results obtained by Jeske et al. [20] in cancer patients treated with chemotherapy, while lower than that observed in kidney transplant patients from Pakistan and with HIV/AIDS individuals from Ethiopia, at 53% and 43.6%, respectively [21,22] (Table 3).

Phulsunge et al. [15] showed that during intensive chemotherapy, patients with HM have a prevalence of intestinal parasites that can be arranged in the following descending series: ALL < AML < PCM and lymphoma ~69%; ~55%; ~47%, respectively. Taşova et al. [26] detected parasitic infections in ~27% of hematology patients undergoing chemotherapy with neutropenia. In hemato-oncological patients, *Blastocystis* spp. and *E. coli* were reported in ~13% and 1.5%, respectively. The presence of *Blastocystis* spp. was most commonly confirmed in patients with AML, ALL, CML, CLL and NHL, while *E. coli* was noted in patients with AML, ALL and CML.

Patients with marked T-cell deficiencies do not exhibit increased susceptibility to giardiasis [18]. Some authors observed that the weighted prevalence of *G. intestinalis* in patients with cancer did not exceed 7% [26]. In this study, we found *G. intestinalis* cysts and confirmed coproantigen in a patient with DLBCL, but this parasite was not a common opportunistic infection in patients with cancer or cancer chemotherapy-induced immunosuppression, suggesting that not all forms of immunosuppression predispose to the infection [18]. Mahdavi et al. [37] demonstrated that the immunodeficiency status of cancer patients is a possible risk factor for acquiring *Giardia* infection, and they observed that the weighted prevalence of giardiasis was higher among patients suffering from HMs compared with colorectal patients as well as those individuals with mixed cancers.

Reduced immunity in HM patients is manifested clinically by chronic and increasing infections or, less commonly, autoimmunity. The course of opportunistic parasitic infection may be atypical, severe, prolonged and with resistance to antibiotic therapy [8]. It has been reported that patients undergoing hemato-oncological treatment report gastrointestinal symptoms [4]. Moreover, gastrointestinal symptoms occurring during parasitic infections are similar to symptoms after chemotherapy and include weight loss, loss of appetite and diarrhea. This may lead to misdiagnosis. In this study, there was no association between the presence of parasites and intestinal symptoms. Some authors suggest that, during hematological therapy, patients do not report gastric symptoms due to the immunosuppression which delays the inflammatory response [15]. Abdominal pain, diarrhea, vomiting and nausea have been reported in hemato-oncological patients with blastocystosis. Although some *Blastocystis* spp. infections indicate the pathogenic nature of this protozoan, usually they are asymptomatic [15]. Tan [38] observed that a large number of parasites in the intestine (>5 parasites per high-power field) entails gastrointestinal symptoms. In this study, all those infected with *Blastocystis* spp. from the HM group and the CG had less than five vacuolar forms per high-power field.

It has been shown that *Blastocystis* spp. can induce the process of caspase-dependent apoptosis without changes in the intestinal epithelium of rats, while at the same time maintaining its correct functioning and the impermeability of the barrier [39]. Kumarasamy et al. [40] suggested that *Blastocystis hominis* should be considered an opportunistic parasite, e.g., when the parasite is confirmed in colorectal cancer during chemotherapy.

*Cryptosporidium* spp. is one of the major causes of diarrhea in HIV-positive patients [5,41]. Infection in immunocompetent patients is either asymptomatic or presents with profuse acute or persistent watery diarrhea, nausea and vomiting, stomach cramps and occasionally a fever that lasts approximately two weeks. Full-blown invasive infections, which include severe diarrhea (12–17 L per day), gastrointestinal symptoms and weight loss, occur in patients with severely low immunity and can lead to death in HIV-positive patients. In this group of patients, chronic *Cryptosporidium* spp. infection did not develop until T-lymphocyte (CD4) counts were maintained at >180 cells/mL, and 87% of HIV-positive patients with lower lymphocyte counts developed a full-blown chronic infection [42]. We found *Cryptosporidium* spp. in a patient with HIV and BL during immunochemotherapy.

Gastrointestinal parasites were found to be more prevalent in patients with gastrointestinal cancer than in patients with hematological malignancies, lung cancer and/or breast cancer [43]. Some researchers have suggested that the chronic presence of *Cryptosporidium* spp. in the gastrointestinal tract of the host can induce pathological changes of an adenocarcinoma-like nature. Furthermore, it has been reported that, at low infestation, this opportunistic protozoan can induce gastrointestinal neoplasia [44]. SCID mice were infected with the *Cryptosporidium* strain isolated from patients with lymphoblastic leukemia after bone marrow transplantation in an experimental model. Symptomatic cryptosporidiosis and neoplasm in the stomach, intestine and biliary tract were observed in the animals, and the presence of *Cryptosporidium* spp. was noted in the range of neoplastic lesions [42]. *Cryptosporidium* spp. leads to the activation of nuclear factor kappa B *(NF*-*κB)*, known to induce anti-apoptotic mechanisms and also to transmit oncogenic signals to epithelial cells [42]. Sulżyc-Bielicka et al. [32] reported no significant association between *Cryptosporidium* spp. infection and the tumor grade according to the Astler-Coller scale, but *Cryptosporidium* coproantigen was significantly more prevalent in patients with colorectal cancer before oncological treatment than in a control group without malignant changes. In the two hemato-oncological patients in this study, *Cryptosporidium* spp. and *Blastocystis* spp. were found to co-occur with sigmoid tumors and clear cell renal cell carcinoma.

Chemotherapies other than corticosteroids appear to predispose patients towards parasitic infections more than any other form of anticancer treatment. Cryptosporidiosis has been reported in cases of acute leukemia and as a complication of cancer chemotherapy [16]. In this study, we observed cryptosporidiosis in patients treated with chemotherapeutic agent immunosuppressive drugs (dexamethasone) and aggressive immunochemotherapies: GMALL, PALG and Rhyper-CVAD. Taşova et al. [26] suggested that in patients with HM and intestinal symptoms, *Blastocystis* spp. should be excluded even during intensive chemotherapy treatment, as was confirmed by a patient with ALL in our study, in whom blastocystosis was noted after the first cycle of PALG ALL6 consolidation treatment with methotrexate and dexamethasone.

In immunocompromised patients, mainly with HIV infection, several studies investigated the specific risk factors for this high-risk group and found that drinking tap water from an untreated supply, exposure to animals, unsafe sexual activity and the use of public toilets are associated with *Cryptosporidium* spp. infection. The infection is spread from person to person, from animals, via food and by water [45,46,47]. In patients with HM, the risk factors affecting the occurrence of enteric parasites we found included working in the garden without protective gloves and contact with animals, as was confirmed by Izadi et al. [47]. Gedle et al. [48] in patients with HIV/AIDS from Ethiopia showed that contact with animals, drinking tap water, malnutrition and low number of CD4 T cells are predisposing factors to parasitic diseases in patients with low immunity. Similarly, Jeske et al. [20] showed a relationship between dog and cat ownership and the frequency of intestinal parasitosis, especially giardiasis and cryptosporidiosis. Kindie and Bekele [46] reported no association with diet (e.g., eating unwashed fruits, vegetables and meat) and the presence of enteric parasites in patients with immune deficiency, which was consistent with this study.

In our study, we did not find any correlation between intestinal parasites and travel history, but two patients, who declared a trip to Israel and Kenya (before hospitalization), had protozoan parasites. Cancer patients are at higher risk of infections during neutropenia after chemotherapy, and most patients should be discouraged from traveling during that period [49]. According to the Centers for Disease Control and Prevention, as mentioned by Mikati et al. [50], travelers are considered immunocompromised for three months after their last aggressive treatment. An extended diagnosis of any cases of chronic diarrhea after a return from intertropical and subtropical countries is encouraged, especially in immunocompromised HIV-positive patients, among others, and patients undergoing hemato-oncological treatment [28,51].

## 5. Conclusions

Chemotherapy in patients with hematological malignancies, mainly those with plasma cell myeloma and diffuse large B-cell lymphoma, results in an increased risk of intestinal parasitic infections including opportunistic infections. A number of environmental factors, including pet ownership and contact with soil while gardening without gloves, seem to increase the risk of intestinal parasitosis in patients with hematological malignancies. In our study, we confirmed the infection of *Giardia intestinalis* and *Blastocystis* spp. in HM patients. It is possible that, similar to *Cryptosporidium* spp., these parasites act as opportunistic pathogens in patients undergoing chemotherapy, but this is still an issue that requires further comprehensive research.

Every disturbance of the immune system (e.g., immunosuppression, chemotherapy and HIV) has an influence on the course of the infection; thus, parasitological examination of stools of symptomatic and asymptomatic infections, also during treatment with chemotherapeutics, is advisable.

## Figures and Tables

**Table 1:** Prevalence of intestinal parasites in immunosuppressed patients with hematological malignancies (HMs) and immunocompetent participants (CG) (-, not detected).

Species	HM (*n* = 50)		CG (*n* = 50)	
	*n*	%	*n*	%
*Giardia intestinalis*	1	2.0	-	-
*Cryptosporidium* spp.	5	10.0	-	-
*Blastocystis* spp.	1	2.0	3	6.0
*Entamoeba coli*	1	2.0	-	-
Total	8	16.0 *	3	6.0 *

* *p* < 0.05 for the significance of the difference vs. the control (Mann–Whitney U test).

**Table 2:** The prevalence of intestinal parasites (%) in the HM and CG groups according to environmental risk factors.

	HM (*n* = 50)		CG (*n* = 50)	
	Infected (*n* = 8)	Uninfected (*n* = 42)	Infected (*n* = 3)	Uninfected (*n* = 47)
Drinking tap water				
Yes	25.0	23.8	0	17.95
No	75.0	76.2	100	82.05
Eating unwashed fruits and vegetables				
Yes	37.5	33.3	0	28.21
No	62.5	66.7	100	71.79
Eating raw meat, sea food and fish				
Yes	37.5	31.0	0	43.59
No	62.5	69.0	100	56.41
Gardening without gloves				
Yes	87.5 *	54.8	33.3	20.51
No	12.5 *	45.2	66.7	79.49
Contact with domestic and wild animals				
Yes	75.0 *	54.8	100	56.41
No	25.5 *	45.2	0	43.59
Travel history				
Yes	25.0	16.7	0	42.6
No	75.0	83.3	100	57.4

* *p* < 0.05 for the significance of the difference vs. the control (Mann–Whitney U test).

**Table 3:** The occurrence of intestinal parasites in humans from different part of the world according to immunological status.

Species	Country	Diseases	Number of Patients	Number of Cases	Prevalence, %	Source
*Blastocystis* spp.	Poland	Acute lymphoblastic leukemia	50	1	2	This study
		colorectal cancer	107	13	12.15	[23]
		Immunodeficiency primary/acquired	237	3	1.2	[5]
	Turkey	Cancer	201	29	14.4	[24]
		Cancer	232	25	10.78	[25]
		Leukemia	91	3	3.3	[4]
		Hematological malignancies	206	23	13.1	[26]
		Cancer	85	13	16.2	[27]
	Iran	HIV/AIDS, cancer/solid transplantation	265	11	4.2	[28]
	Saudi Arabia	Colorectal cancer	218	50	23	[29]
	Egypt	Leukemia, lymphoma	145	31	21.4	[30]
*Entamoeba coli*	Poland	Myelodysplastic/myeloproliferative neoplasm	50	1	2	This study
	Turkey	Hematological malignancies	206	3	1.5	[26]
	Egypt	Leukemia, lymphoma	145	16	11.0	[30]
	Brazil	Cancer	73	23	31.1	[20]
*Giardia intestinalis*	Poland	Diffuse large B-cell lymphoma	50	1	2	This study
		Immunodeficiency primary/acquired	237	2	0.7	[5]
	Mexico	Acute lymphoblastic leukemia	77	2	2.6	[31]
	Iran	Cancer including lymphoma	85	2	2.3	[27]
*Cryptosporidium* spp.	Poland	Plasma cell myeloma, acute myeloblastic leukemia, diffuse large B-cell lymphoma, Burkitt’s lymphoma	50	5	10	This study
		Immunodeficiency primary/acquired	237	4	1.4	[5]
		Colorectal cancer	108	14	13	[32]
	Turkey	Leukemia	91	6	6.6	[4]
	India	Cancer	560	7	1.3	[33]
		Malnutrition, HIV/AIDS, cancer	170	2	4.7	[33]
		Hematological malignancies	101	3	3.0	[34]
	China	Gastrointestinal cancers	195	26	13.33	[35]
		Colorectal cancer	116	20	17.24	
		Gastric cancer	51	2	4	
	Pakistan	Kidney transplantation	644	343	53	[20]
	Egypt	Leukemia, lymphoma	124	44	35.5	[30]
	Ethiopia	HIV/AIDS	131	82	43.6	[36]
	Brazil	Cancer	73	13	13.3	[20]
	Mexico	Acute lymphoblastic leukemia	77	7	9.09	[31]
	Columbia	Hematological malignancies HIV/AIDS	111	4	3.6	[6]

## Data Availability

The data sets used and/or analyzed during the current study are available from the corresponding author upon reasonable request.

## Abbreviations

AML, acute myeloblastic leukemia; ALL, acute lymphoblastic leukemia; BL, Burkitt’s lymphoma; CLL, chronic lymphocytic leukemia; DLBCL, diffuse large B-cell lymphoma; EBV, Epstein–Barr virus; CG, control group; GMALL, German Multicenter Adult ALL Study Group; HMs, hematological malignancies; MPN, myelodysplastic/myeloproliferative neoplasm; NHL, non-Hodgkin’s lymphoma; PCM, plasma cell myeloma.
